# Supplementary material for: Structural insights into the mycobacteria transcription initiation complex from analysis of X-ray crystal structures
Source: Nat Commun. 2017 Jul 13;8:16072. doi: 10.1038/ncomms16072 (PMC5511352; doi:10.1038/ncomms16072)
Supplement: Supplementary Information [file ncomms16072-s1.pdf]

File Name: Supplementary Information
Description: Supplementary Figures, Supplementary Tables and Supplementary References

File Name: Peer Review File
Description:

Supplementary Table 1. Table of crystallographic statistics.

	Msm RbpA/RPo[a]
**Data collection**	
Space group	$P2_1$
Combined datasets	1
Cell dimensions	
a, *b*, *c* (Å)	132.06, 163.56, 139.96
α, β, γ (°)	90.00, 107.90, 90.00
Resolution (Å)	50.00 – 3.20 (3.31 – 3.20)[b]
R_{merge}[c]	0.220 (1.751)
R_{pim}[c]	0.141 (1.300)
$\langle I \rangle/\sigma I$	7.48 (0.63)
CC1/2[d]	0.985 (0.153)
CC*[d]	0.996 (0.516)
Completeness (%)	95.1 (76.6)
Redundancy	3.3 (2.3)
**Refinement**	
Resolution (Å)	50.00 – 3.20 (3.31 – 3.20)
Total reflections	291,359 (15,032)
Unique reflections	88,065 (6,119)
R_{work} / R_{free}	0.2533/0.2798 (0.3672/0.3853)
CC_{work}/CC_{free}[c]	0.935/0.897 (0.329/0.201)
No. atoms	26,557
Macromolecules	26,515
Ligand/ion	34
Water	6
B-factors	
Macromolecules	88.10
Ligand/ions/water	105.00
R.m.s deviations	
Bond lengths (Å)	0.002
Bond angles (°)	0.46

[a] One crystal was used for data collection.
[b] Values in parentheses are for highest-resolution shell.
[c] ref. [1]
[d] ref. [2]

Wavelength (Å)	0.97918
Wilson B-factor (Å2)	85.84
Total reflections	291,359 (15,032)
Unique reflections	88,065 (6,119)
Ramachandran favored (%)	94
Ramachandran outliers (%)	0.71
Clashscore	20.81

Supplementary Table 2. Conservation of *Eco* $\sigma^{70}_{1.1}$ in bacterial clades.

phylum	Phylum/class/family	taxid	BLAST hits[a]		%
			Eco $\sigma^{70}_{1.2\text{-}4.2}$ [Residues 375-613]	*Eco* $\sigma^{70}_{1.1}$ [residues 1-95]	($\sigma^{70}_{1.1}$:$\sigma^{70}_{1.2\text{-}4.2}$)
proteobacteria	γ-proteobacteria	1236	3,991	3,546	89
	β-proteobacteria	28216	2,185	1,743	80
	ζ-proteobacteria (mariprofundales)	580370	7	5	71
	α-proteobacteria	28211	1,993	1,736	87
	magnetococcus	162171	1	1	100
	δ-proteobacteria	28221	449	293	65
	ε-proteobacteria	29547	727	0	0
	Nitrospiraceae	189779	39	19	49
	Aquificaceae	64898	18	9	50
	Acidobacteria	57723	80	63	79
	spirochaetes	203691	362	135	37
	Deinococcus-thermus	1297	89	0	0
	thermotogae	200918	53	41	77
	fusobacterium	848	110	34	31
Firmicutes	Mollicutes	31969	215	6	3
	lactobacillales	186826	1,125	227	20
	Bacillales	1385	1,012	735	73
	Clostridia	186801	1,649	602	37
	actinobacteria	201174	1,467	94	6
	chloroflexi	200795	206	60	29
	cyanobacteria	1117	1,075	2	0.2
	bacteroidetes	976	954	2	0.2
	chlorobi	1090	21	0	0
	chlamydiae	204428	66	53	80
	planctomycetes	203682	191	57	30

[a] Search database Non-redundant protein sequences (nr) using Blastp (protein-protein BLAST); searches restricted to Entrez query ‘rpoD’; expect threshold = 0.1; word size = 2; matrix = BLOSUM62; gap costs, existence: 13, extension: 1; conditional compositional score matrix adjustment.

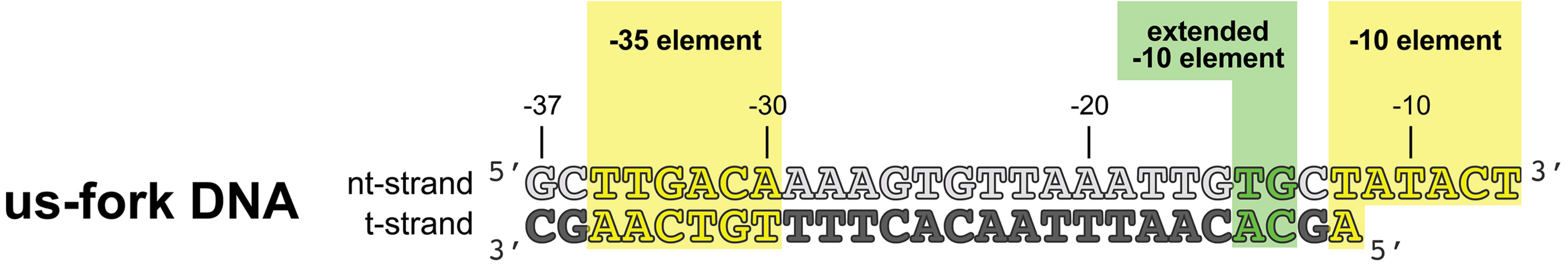

Supplementary Figure 1

Supplementary Figure 1 | Us-fork promoter DNA fragment. Sequence of the us-fork promoter fragment used in the 2.76 Å-resolution *Msm* TIC structure [3]. The numbers above denote the DNA position with respect to the transcription start site (+1). The DNA sequence is derived from the full con promoter [4]. The nt-strand DNA (top strand) is colored light gray; the t-strand DNA (bottom strand), dark grey. The -35 and -10 elements are shaded yellow. The extended -10 [5] is colored green.

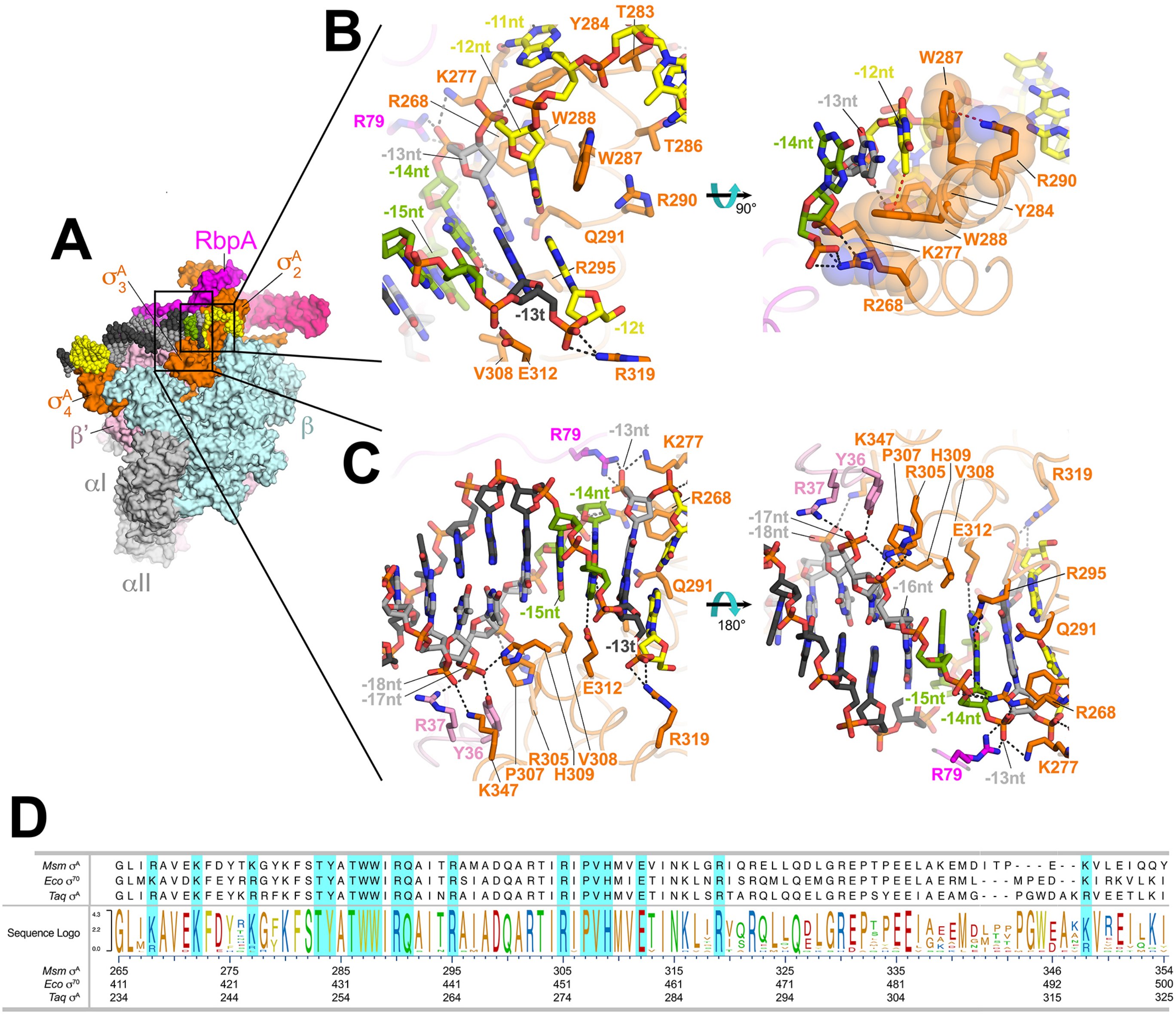

Supplementary Figure 2

Supplementary Figure 2 | Details of Protein/DNA interactions in the *Msm* TIC.

A. Overall view of the *Msm* TIC structure (PDB ID 5TW1) [3], color-coded as in Fig 1B. Proteins are shown as molecular surfaces. The nucleic acids are shown in CPK format. The boxed regions are magnified in **B** and **C** to the right.

B. Magnified view showing the upstream ds/ss junction of the transcription bubble. Side chains of σ^A (orange) and RbpA (magenta, R79) residues that interact with the DNA are shown (polar interactions are shown as dashed grey lines). (right) top view. (left) side view, showing the 'chair' conformation of the σ^A W-dyad (W287/W288) and supporting Arg residues (shown with transparent CPK spheres).

C. Magnified view showing protein interactions with duplex DNA upstream of the transcription bubble. DNA-interacting side chains of σ^A (orange), RbpA (magenta, R79), and β' (Y36 and R37, pink) are shown.

D. Sequence alignment showing a region of *Msm* σ^A, *Eco* σ^{70}, and *Taq* σ^A (the respective numbering is shown at the very bottom). Below the sequences is shown a sequence logo [6] derived from an alignment of more than 1,000 Group 1 σ sequences [7]. Key DNA-interacting residues are shaded blue.

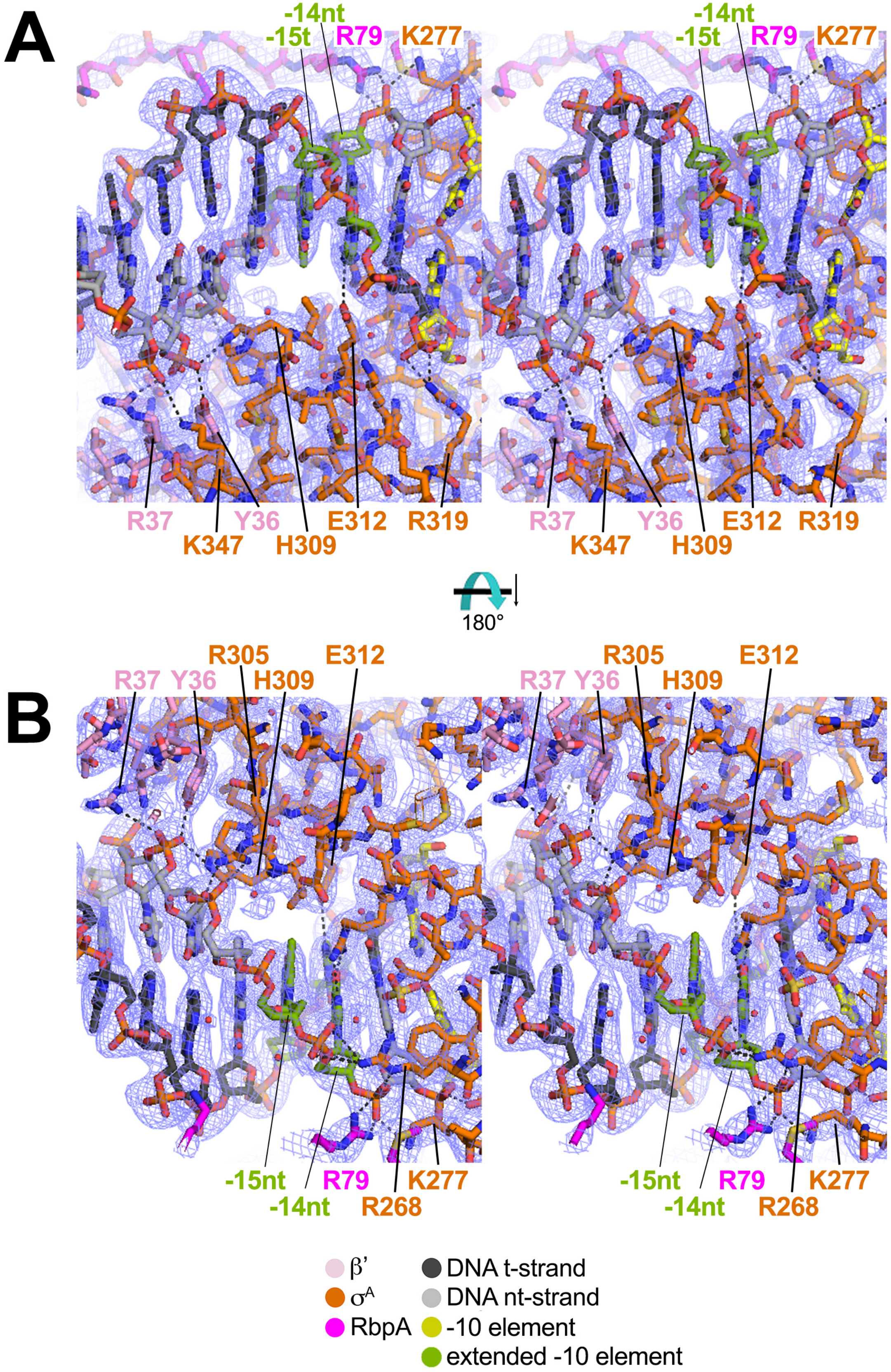

Supplementary Figure 3

Supplementary Figure 3 | Electron density maps showing protein interactions with duplex DNA upstream of the transcription bubble.

A. Stereo view showing the 2.76 Å-resolution $2F_o - F_c$ electron density map (blue mesh, contoured at 1.5σ) from the *Msm* RbpA/Eσ^A/us-fork structure (PDB ID 5TW1) [3]. The view is the same as Supplementary Fig. 2C(left).

A. Stereo view showing the 2.76 Å-resolution $2F_o - F_c$ electron density map (blue mesh, contoured at 1.5σ) from the *Msm* RbpA/Eσ^A/us-fork structure (PDB ID 5TW1) [3]. The view is the same as Supplementary Fig. 2C(right).

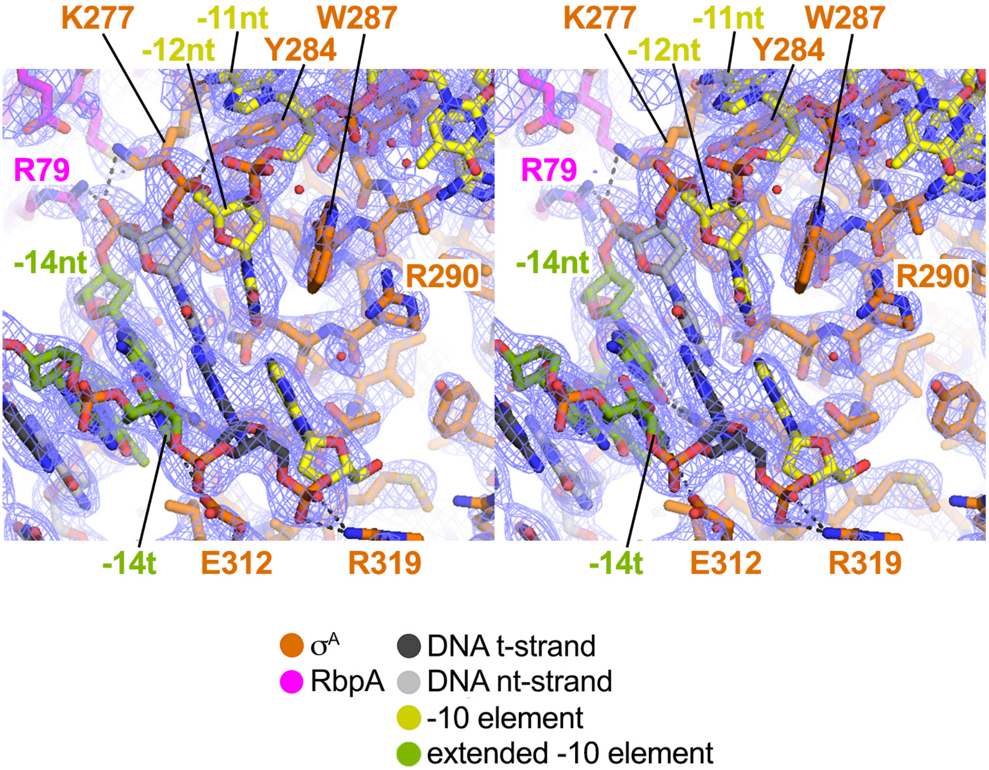

Supplementary Figure 4

Supplementary Figure 4 | Electron density map showing protein interactions with the upstream edge of the transcription bubble. Stereo view showing the 2.76 Å-resolution $2F_o - F_c$ electron density map (blue mesh, contoured at 1.5σ) from the *Msm* RbpA/Eσ^A/us-fork structure (PDB ID 5TW1) [3]. The view is the same as Supplementary Fig. 2B(left).

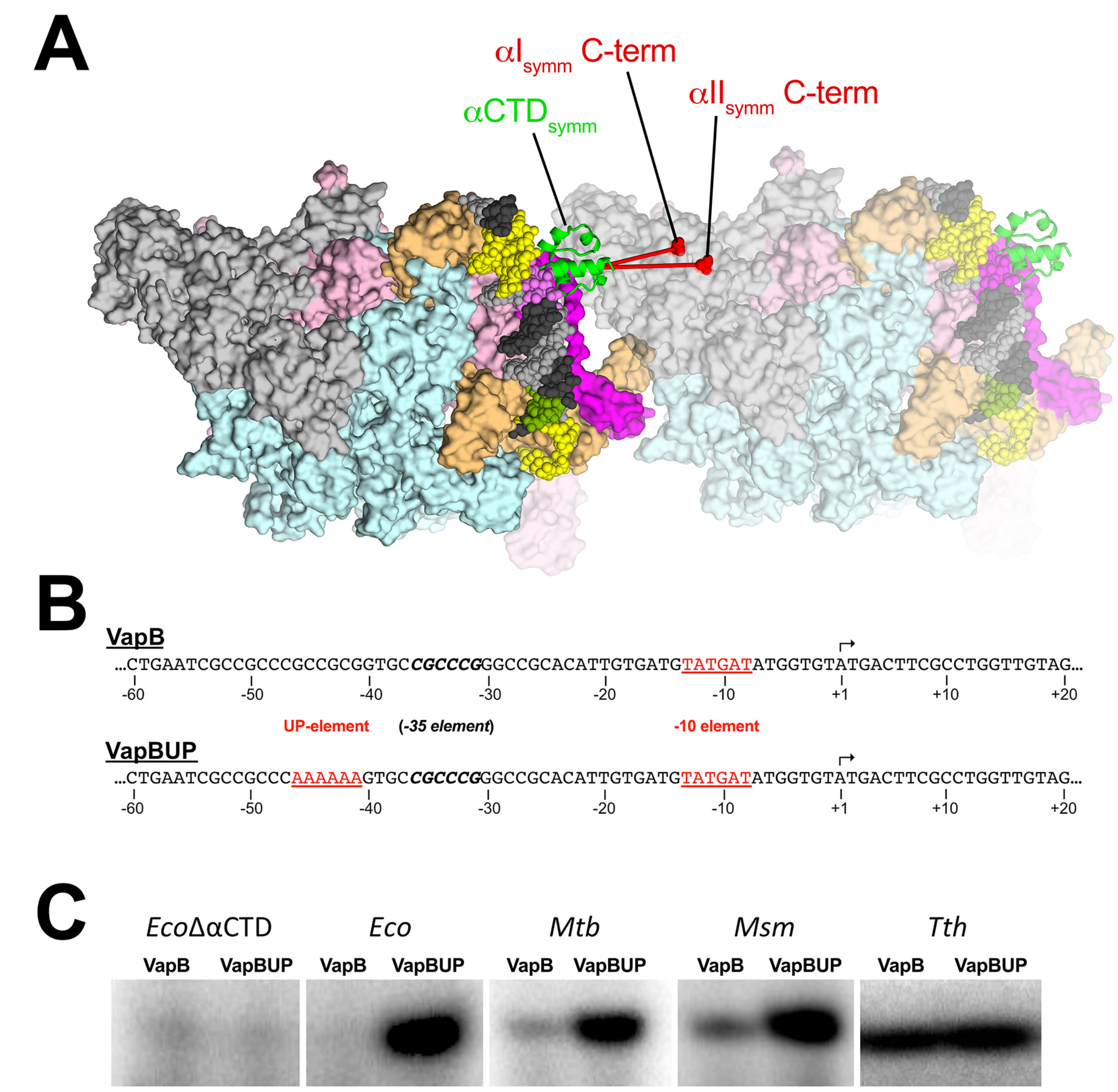

Supplementary Figure 5

Supplementary Figure 5 | *Msm* αCTD.

A. Overall view of the *Msm* TIC structure (PDB ID 5TW1) [3], color-coded as in Fig. 2B. A neighboring symmetry-related complex is also shown (lighter colors). The green αCTD_{symm} could be derived from the symmetry-related αI_{symm} or αII_{symm} (red lines are drawn from the α_{symm} C-termini to the N-terminus of αCTD_{symm}).

B. Sequences of the *Mtb* VapB (top) and engineered VapBUP (bottom) promoter templates. The VapB promoter lacks a recognizable -35 element. The normal position of the -35 element is shown with the bold, italicized sequence.

C. Representative transcription assays showing abortive transcripts (ApUpG) synthesized from VapB and VapBUP by the noted RNAP holoenzymes. These assays were performed in triplicate and the results are quantitated in Fig. 2F.

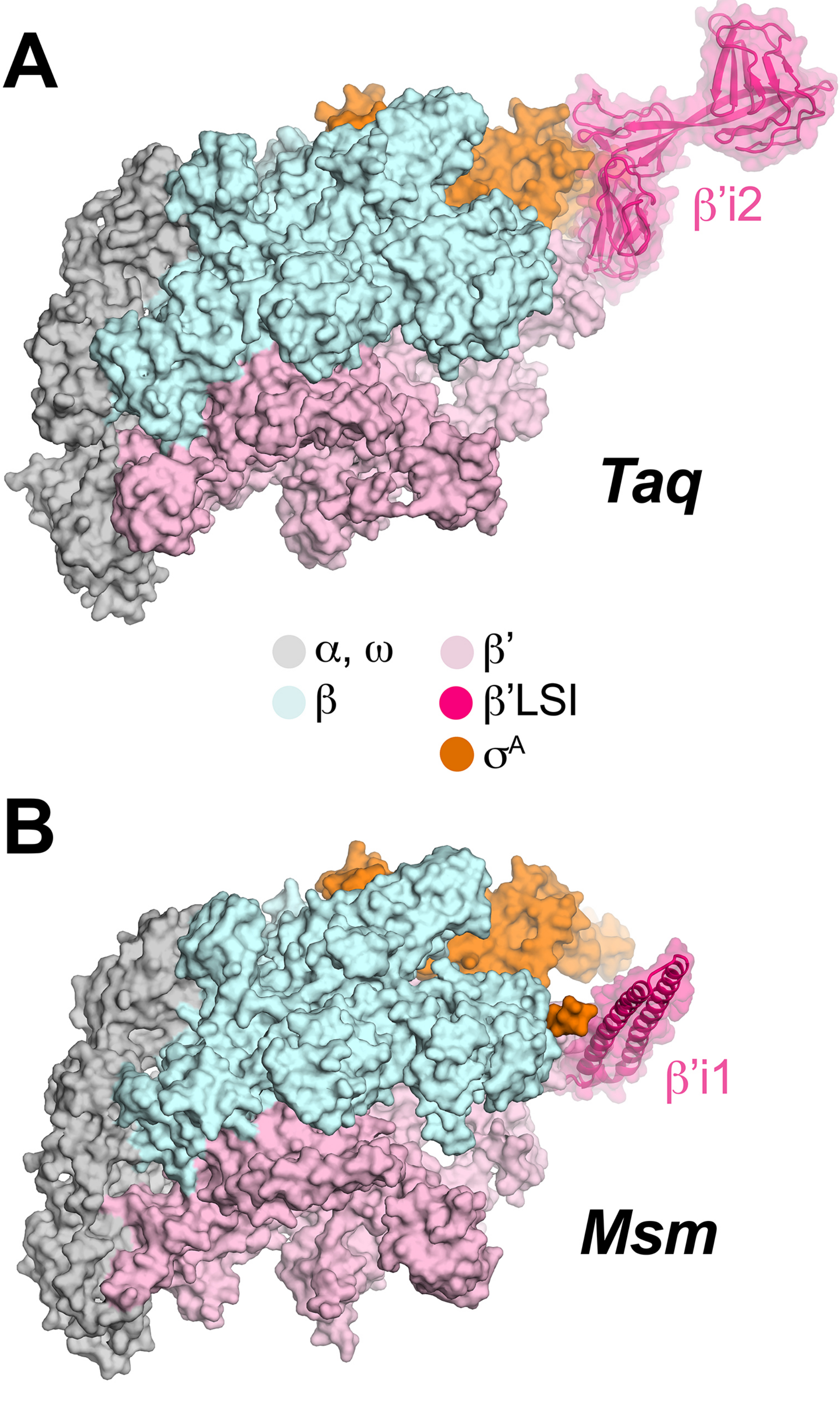

Supplementary Figure 6

Supplementary Figure 6 | *Taq* and *Msm* β'-lineage-specific inserts.

A. *Taq* σ^A-holoenzyme (PDB ID 4XLN with nucleic acids removed) [7], shown as a molecular surface color-coded as shown in the legend. The β'i2 is shown with a transparent molecular surface, revealing the backbone ribbon beneath.

B. *Msm* σ^A-holoenzyme (PDB ID 5TW1 with RbpA and nucleic acids removed) [3], shown as a molecular surface color-coded as shown in the legend. The β'i1 is shown with a transparent molecular surface, revealing the backbone ribbon beneath.

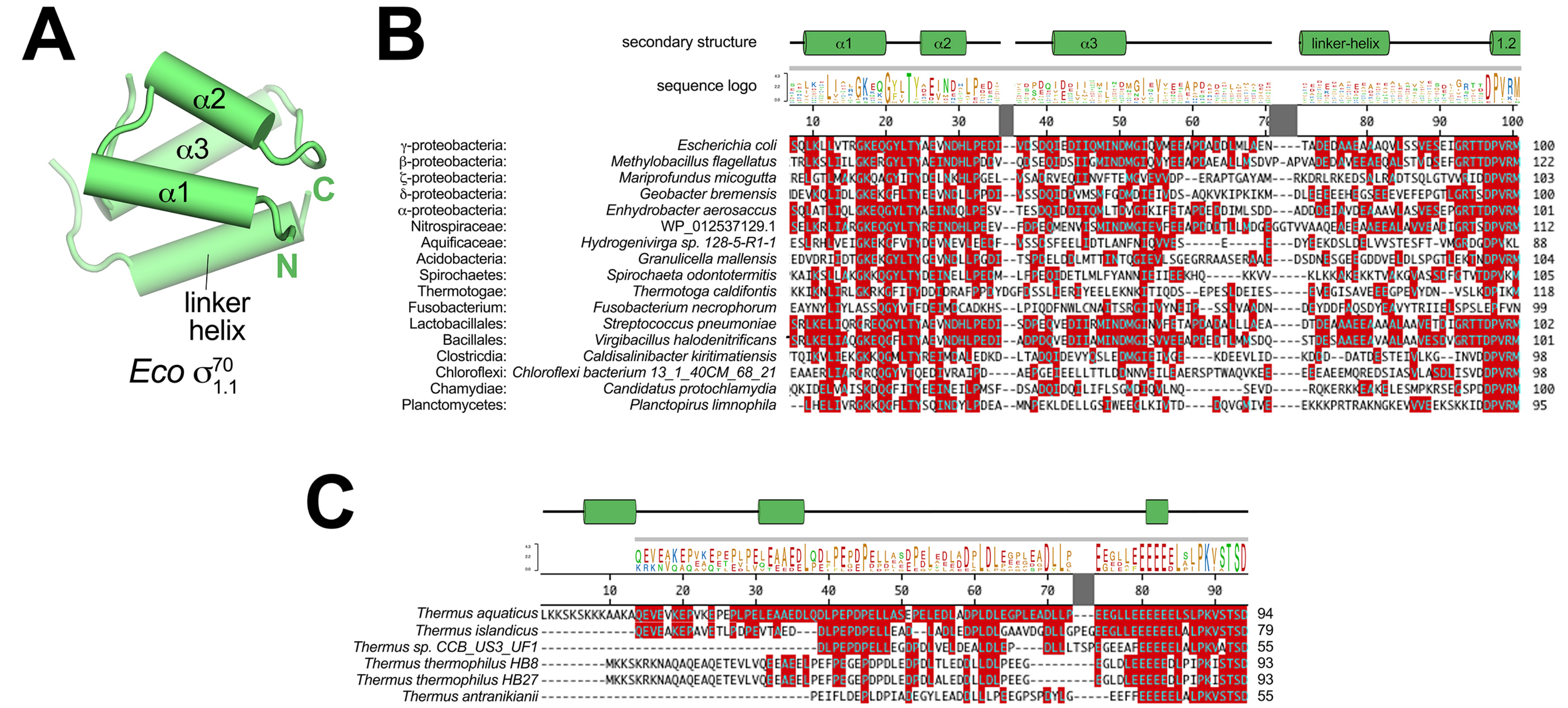

Supplementary Figure 7

Supplementary Figure 7 | *Eco* σ^{70} and *Thermus* σ^{A} N-terminal extensions.

A. Structure of *Eco* $\sigma^{70}_{1.1}$ and $\sigma^{70}_{1.1\text{-}1.2\ \text{linker}}$ (taken from PDB ID 4LK1) [8].

B. Sequence alignment of *Eco* σ^{70}_{N} (residues 1-95) with representative σ^{A}_{N} sequences from each of the clades with high representation of *Eco* σ^{70}_{N} homologs (see Supplementary Table 2). The numbering scale on top denotes *Eco* σ^{70} numbering. Residues conserved in more than half the sequences are shaded red. The sequence logo [6] is derived from the alignment shown. Above the sequence logo is shown the secondary structure of *Eco* σ^{70}_{N} (PDB ID 4LK1) [8].

C. Sequence alignment of *Taq* σ^{A}_{N} (residues 1-94) with representative deinococcus-thermus σ^{A}_{N} sequences. The numbering scale on top denotes *Taq* σ^{A} numbering. Residues conserved in more than half the sequences are shaded red. The sequence logo [6] is derived from a slightly larger alignment containing 9 sequences. Above the sequence logo is shown the predicted secondary structure [9] of *Taq* σ^{A}_{N}.

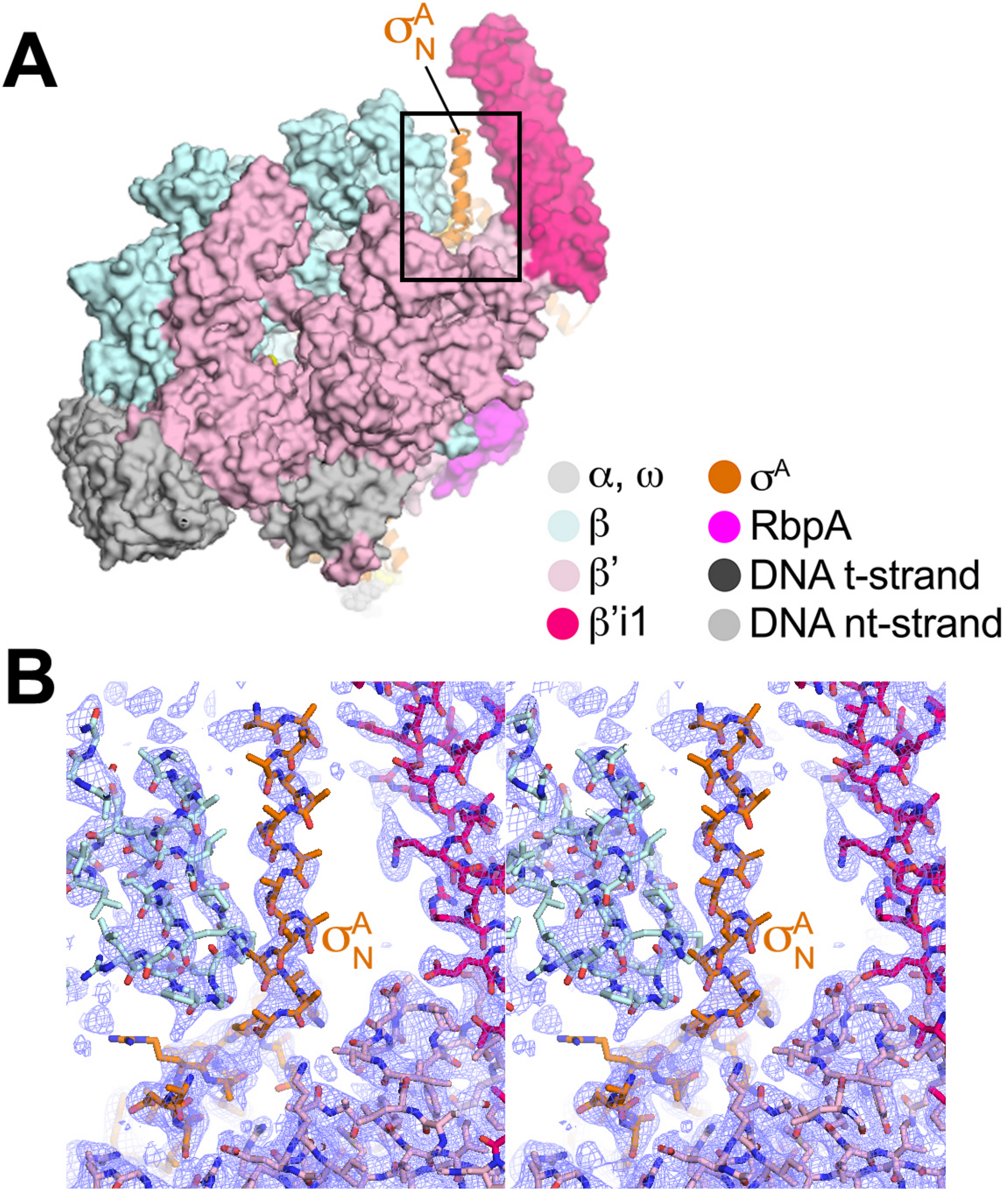

Supplementary Figure 8

Supplementary Figure 8 | Electron density map showing the σ^A_N-helix.

A. Overall view of the *Msm* TIC structure (PDB ID 5TW1) [3], color-coded as shown in the legend. Proteins are shown as molecular surfaces except σ^A is shown as a backbone ribbon. The boxed region is magnified in Supplementary Fig. 9B below.

B. Stereo view showing the boxed region from Supplementary Fig. 9A (above). The 2.76 Å-resolution $2F_o - F_c$ electron density map (blue mesh, contoured at 1.5σ) is shown from the *Msm* RbpA/Eσ^A/us-fork structure (PDB ID 5TW1) [3].

Supplementary references

1. Diederichs, K. & Karplus, P. A. Improved R-factors for diffraction data analysis in macromolecular crystallography. *Nat Struct Biol* **4,** 269–275 (1997).

2. Karplus, P. A. & Diederichs, K. Linking Crystallographic Model and Data Quality. *Science* **336,** 1030–1033 (2012).

3. Hubin, E. A. *et al.* Structure and function of the mycobacterial transcription initiation complex with the essential regulator RbpA. *Elife* **6,** e22520 (2017).

4. Gaal, T. *et al.* Promoter recognition and discrimination by EsigmaS RNA polymerase. *Molecular Microbiology* **42,** 939–954 (2001).

5. Keilty, S. & Rosenberg, M. Constitutive function of a positively regulated promoter reveals new sequences essential for activity. *J. Biol. Chem.* **262,** 6389–6395 (1987).

6. Schneider, T. D. & Stephens, R. M. Sequence logos: a new way to display consensus sequences. *Nucl Acids Res* **18,** 6097–6100 (1990).

7. Bae, B., Feklistov, A., Lass-Napiorkowska, A., Landick, R. & Darst, S. A. Structure of a bacterial RNA polymerase holoenzyme open promoter complex. *Elife* **4,** e08504 (2015).

8. Bae, B. *et al.* Phage T7 Gp2 inhibition of Escherichia coli RNA polymerase involves misappropriation of σ70 domain 1.1. *Proceedings of the National Academy of Sciences* **110,** 19772–19777 (2013).

9. Rost, B., Yachdav, G. & Liu, J. The PredictProtein server. *Nucl Acids Res* **32,** W321–6 (2004).
